# Rapid Evaluation of Novel Therapeutic Strategies Using a 3D Collagen-Based Tissue-Like Model

**DOI:** 10.3389/fbioe.2021.574035

**Published:** 2021-02-16

**Authors:** Pauline Maury, Erika Porcel, Adrien Mau, François Lux, Olivier Tillement, Pierre Mahou, Marie-Claire Schanne-Klein, Sandrine Lacombe

**Affiliations:** ^1^Université Paris-Saclay, CNRS, Institut des Sciences Moléculaires d’Orsay, Orsay, France; ^2^Institut Universitaire de France, Paris, France; ^3^Institut Lumière Matière, Université Claude Bernard Lyon 1, UMR 5306 CNRS-UCBL, Villeurbanne, France; ^4^Laboratoire d’Optique et Biosciences, Ecole Polytechnique, Centre National de la Recherche Scientifique, Institut National de la Santé et de la Recherche Médicale, Institut Polytechnique de Paris, Palaiseau, France

**Keywords:** 3D cell model, collagen, hydrogel, nanoagents, radioenhancement, internalization, radiosensitization, SHG

## Abstract

2D cell cultures are commonly used to rapidly evaluate the therapeutic potential of various treatments on living cells. However, the effects of the extracellular matrix (ECM) including the 3D arrangement of cells and the complex physiology of native environment are missing, which makes these models far from *in vivo* conditions. 3D cell models have emerged in preclinical studies to simulate the impact of the ECM and partially bridge the gap between monolayer cultures and *in vivo* tissues. To date, the difficulty to handle the existing 3D models, the cost of their production and their poor reproducibility have hindered their use. Here, we present a reproducible and commercially available “3D cell collagen-based model” (3D-CCM) that allows to study the influence of the matrix on nanoagent uptake and radiation effects. The cell density in these samples is homogeneous. The oxygen concentration in the 3D-CCM is tunable, which opens the opportunity to investigate hypoxic effects. In addition, thanks to the intrinsic properties of the collagen, the second harmonic imaging microscopy may be used to probe the whole volume and visualize living cells in real-time. Thus, the architecture and composition of 3D-CCMs as well as the impact of various therapeutic strategies on cells embedded in the ECM is observed directly. Moreover, the disaggregation of the collagen matrix allows recovering of cells without damaging them. It is a major advantage that makes possible single cell analysis and quantification of treatment effects using clonogenic assay. In this work, 3D-CCMs were used to evaluate the correlative efficacies of nanodrug exposure and medical radiation on cells contained in a tumor like sample. A comparison with monolayer cell cultures was performed showing the advantageous outcome and the higher potential of 3D-CCMs. This cheap and easy to handle approach is more ethical than *in vivo* experiments, thus, giving a fast evaluation of cellular responses to various treatments.

## Graphical Abstract

3D cell collagen-based model with tunable environment conditions (oxygen concentration, nanodrug exposure or irradiations).

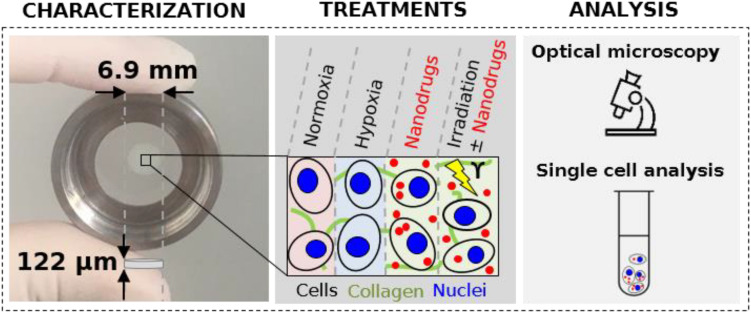

## Introduction

*In vitro* cell models are commonly used to study the cause and progression of diseases, to predict treatment effectiveness and to analyze drug-induced toxicities (Magdeldin et al., 2014; Bell et al., 2016; Rayner et al., 2019). Monolayer cultures have led to numerous advances even if success at the bench does not always translate into success at the bedside. Despite their accessibility and low cost, the current 2D models do not faithfully mimic *in vivo* tissue conditions (Achilli et al., 2012; Lazzari et al., 2017). Simple spatial organization appears to be a major drawback: the predominance of the cell-surface attachment allows the cells to spread which modifies their morphology and behavior (Eke, 2011). In addition, in 2D cultures, cells are bathed in a standard cell culture medium whose composition differs from that of the cellular microenvironment. *In vivo*, cells are surrounded by a natural structure, the extracellular matrix (ECM), which acts as a structural and biochemical support. The ECM, which is primarily composed of water, proteins and polysaccharides, provides a mechanical framework that influences cell shape, stiffness and adhesion (Pedersen and Swartz, 2005; Ghosh et al., 2007; Qiu et al., 2017). The ECM also permits communication between cells via the transmission of biochemical signals and plays a crucial role in the regulation of numerous cell functions such as proliferation, differentiation and gene expression (Even-Ram and Yamada, 2005; Walker et al., 2018). These essential communication pathways involving the ECM-cell and cell-cell interactions cannot be reproduced in 2D cultures. Finally, in 2D monolayers cells have direct access to molecular oxygen. In this case, mass transport and diffusion phenomena are too simplistic and not representative of the various conditions encountered in the cytoarchitecture of native tissues.

In this perspective, 3D cell models have been developed to overcome the limitations of the current 2D models. Even if all aspects of the microenvironment cannot be fully captured such as vascularization and circulation processes, reticuloendothelial and hepatic uptakes or local immune repose, *in vitro* 3D models recreate numerous features of living cells. The presence of an ECM makes the models more architecturally and physiologically relevant and allows a more realistic evaluation of the cell response (Achilli et al., 2012; Benien and Swami, 2014; Costa et al., 2016; Nath and Devi, 2016; Cui et al., 2017; Lazzari et al., 2017; Ryu et al., 2019). For instance, when cells are irradiated in 2D and 3D models, substantial differences in the DNA damage response are observed (Sedelnikova et al., 2007; Storch et al., 2010; Suzuki et al., 2010; Zhao et al., 2015), specifically in cell cycle arrest (Topsch et al., 2007; Walenta and Mueller-Klieser, 2016) and repair kinetics (Asaithamby et al., 2011; Acheva et al., 2014).

In the field of nanotoxicity, Belli and coworkers (Belli et al., 2017) showed that monolayer cultures are not fully suitable to study the nanoparticles (NP) internalization because the membrane area in contact with NPs is smaller than for 3D cells. The adhesion of the cells to the plastic substrate induces a reduction of the exposure area. In 3D, the surface of interaction is higher because only the parts of cells contact cannot be exposed to NPs. Moreover, in the absence of ECM, NPs interact directly with the cell membrane, which may modify their uptake. So, 3D-CCMs better reproduce the microenvironment of NPs interacting with cells. Finally, drastic modifications in cytoskeletal arrangement and cell membrane tension were observed in cells cultured in 2D models. This effect, avoided in the case of 3D samples, strongly influences internalization pathways (Storch et al., 2010).

There is therefore an undeniable interest to model nanoagents diffusion in the presence of the ECM to better predict *in vivo* therapeutics behavior (Leeman et al., 2002; Anderson et al., 2006; Gomez-Roman et al., 2016). Currently, two types of 3D cellular models have emerged: (i) scaffold-based 3D models (i.e., hydrogels) and (ii) non-scaffold-based 3D models (i.e., spheroids) (Lazzari et al., 2017; Ferreira et al., 2018; Chaicharoenaudomrung et al., 2019). In spheroids, ECM is produced after a period of culture maintenance, whist scaffold-based 3D models dispose of a pre-existing matrix (Caliari and Burdick, 2016; Costa et al., 2016; Ferreira et al., 2018). In this study, collagen was used as matrix because of its biomimetic properties. Biocompatible, biodegradable and non-toxic, it provides a native viscoelastic environment for embedded cells, mimicking physiological conditions (Antoine et al., 2014; Caliari and Burdick, 2016; Dong and Lv, 2016; Curtin et al., 2018; Catoira et al., 2019). The hydrogels produced are less fragile and easier to manipulate than conventional spheroids (Lin and Chang, 2008; Achilli et al., 2012; Vadivelu et al., 2017). In the present work, the robustness and the preparation reproducibility of commercial 3D cell collagen-based models (3D-CCMs) were assessed. In this purpose, the structure and the size of the samples were characterized as well as the viability and the metabolic activity of the cells. By tuning the oxygen concentration in the environment, we probed the capacity of the sample to mimic hypoxia tissues, such as found in highly lethal and radioresistant tumors (Hirayama et al., 2005; Jiang et al., 2011; Wang et al., 2019). Finally, the performance of 3D-CCM as a tissue-mimicking model was probed by studiying the cell uptake and toxicity of gadolinium based NPs as well as NPs impact on radiation effects on HeLa cells.

## Materials and Methods

### Sample Preparation

This 3D model was first implemented using HeLa human cervical adenocarcinoma cells (ATCC^®^ CCL-2^TM^) and most of the experiments were performed with this immortalized cell line. For comparison, primary dermal fibroblasts (ATCC^®^ PCS-201-010^TM^) (human fibroblasts derived from the foreskin of male African newborn with spindle-shaped morphology) were used for 3D-CCM characterization and evaluation of the reproducibility. All the cells were purchased in 2016 from ATCC© (ATCC France, Molsheim, France). Several ampoules containing cells at early passage were then generated and frozen to have a stock. HeLa cells were used until passage 25 and fibroblasts until passage 15 before returning to stock.

#### Cell Culture

Adherent cells were cultured in monolayer in complete medium composed of Dulbecco’s Modified Eagle Medium supplemented with 10% fetal bovine serum, 1% penicillin and 1% streptomycin (Life Technologies^TM^). The cells were plated in T-75 flasks and maintained in an incubator at 37°C and 5% CO_2_. Once cells were confluent, they were harvested using trypsin. Some cells were seeded on a plastic substrate and maintained in monolayer conditions for experimental use in 2D models. The remainder of the cells was collected to prepare 3D-CCMs according to the protocol described below.

#### 3D-CCM Production

3D-CCM was produced using a RAFT^TM^ kit (Lonza©) according to the protocol distributed by the manufacturer (Lonza, 2016).

First, the HeLa cell suspension obtained from the cell culture was centrifuged at 20°C for 7 min at 1,100 rpm and resuspended to obtain an appropriate concentration of 2.4–2.6 × 10^7^ cells/ml, as determined by a Luna (Logos Biosystems©) automated cell counter. This cell solution was homogeneously mixed with 10X Modified Eagle Medium, neutralizing solution and 2 mg/ml rat-tail type I collagen solution (diluted in 0.6% acetic acid) to obtain a mixture of cells embedded in collagen in the liquid phase with a dilution rate of 4.2%. During this process, all liquids were maintained at 4°C and kept on ice to prevent unwanted polymerization. To reduce uncertainty and batch-to-batch variability, all samples used in the same experiment were prepared from the same solution. A volume of 320 μl from this solution was dispensed into each well of a 96-well culture plate to seed around ∼350,000 cells per 3D-CCMs. After 15 min in the incubator, RAFT^TM^ absorbers were finally placed at room temperature on top of each well for 15 min to obtain the hydrogels. Then, 200 μl of fresh complete medium was added to each well after collagen gel formation and 3D-CCMs were maintained in the incubator during 12–36 h prior to the experiment performed.

For model characterization purposes, 3D-CCMs were also prepared with human primary dermal fibroblasts following exactly the same process. These samples were prepared with a cell suspension at a concentration of 5.3 × 10^6^ cells/ml to obtain 72,000 cells per 3D CCM.

The production of this model is a rapid process (requiring approximately 2 h) and a simple procedure that can be developed in conventional biology laboratories.

### Microscopy Methods

Structural characterization of 3D-CCMs was performed using complementary microscopy techniques. Fluorescence techniques (confocal and multiphoton microscopy) were used to image 3D-CCM, while transmission images allowed the visualization of cell morphology on living samples.

#### Confocal Microscopy

Confocal images of 3D-CCMs were acquired with a LEICA SP5 confocal system. Cell nuclei and the plasma membrane were stained for 30 min with a 1 μmol/L Hoescht 33342 solution (exc: 350 nm/em: 461 nm) (Thermo Fisher Scientific©) and Cell Mask^TM^ Deep Red Actin tracking Stain (Invitrogen^TM^) respectively. Data acquisition was performed with a scan speed of 400 Hz. FOV and pixel sizes used were reported in the legend of the figures. For each sample, transmission images were captured together with the fluorescent images.

#### Multiphoton Microscopy

Multiphoton microscopy was the method used to characterize the model structure. This technique has an improved penetration depth within scattering samples relative to confocal microscopy. Thus, 3D images may be recorded along the full depth of the sample. Moreover, multiphoton microscopy provides complementary modes of contrast, notably SHG, which allows specific imaging of fibrillary collagen without any labeling. The combination of SHG and 2PEF thus enables the simultaneous detection of the collagen of the 3D sample and the cell nuclei, without any cross-talk, which results in the availability of multimodal z-stacks for further analysis (Strupler, 2006).

Sequential 3D acquisitions were performed with a commercial multiphoton microscope (TriM Scope II, LaVision BioTec) equipped with two ultrafast oscillators (Mai Tai HP DeepSee, λ = 690–1040 nm, Spectra Physics and Insight DeepSee, λ = 690–1300 nm, Spectra Physics) and a low magnification and high NA microscope objective (25 × 1.05NA, XLPLN25XWMP2, Olympus). Collagen was imaged using second harmonic imaging microscopy derived from a non-linear optic effect termed second harmonic generation (SHG). The signal is generated by a beam of the Insight DeepSee laser set at λ = 1150 nm. The SHG scattered light centered approximately 575 nm was detected by a photomultiplier tube placed in transmission (H7422-40, Hamamatsu) and separated from the laser light by a dichroic mirror (Di02-R635, Semrock) and an interference filter (FF02-575-25, Semrock). Cell nuclei were mapped by two-photon excitation microscopy. Two-photon fluorescence (2PEF) was generated by the fluorophore Hoescht 33342 when irradiated by the laser beam from the MaiTai set at λ = 830 nm after 30 min of staining with a 1 μmol/L Hoescht 33342 solution (Thermo Fisher Scientific©). The signals were collected in epidetection mode by a photomultiplier tube (H6780-01, Hamamatsu) and separated from the laser light by a dichroic mirror (T695lpxr, Chroma) and an interference filter (FF01-450-70 or FF01-460-80, Semrock). Data acquisition was performed on a 350 μm square field of view with a pixel size of 0.192 μm and an acquisition frequency of 400 Hz. Images were captured with a z-step of 1 μm.

#### Image Processing

Two parameters were investigated to determine the reproducibility of the production method: sample thickness and cell distribution. The volume and shape of 3D models are known to be sources of variability that can lead to different treatment responses (Zanoni et al., 2016). Thus, size uniformity is a key parameter that was evaluated from sample thickness measurements. This parameter was calculated from the acquired SHG stacks according to the determination of a z-range containing a detectable collagen signal.

In parallel, we developed a Python code (V. 3.7) to analyze the cell distribution inside 3D-CCMs. Our program is open-access, available online on Zenodo (doi: 10.5281/zenodo.381436) and described in detail in the Appendices, Section A. Briefly, each 2PEF image stacks were thresholded to create binary images. The small areas (<3 pixel × 3 pixel) were removed from the binary image and all cell nuclei contained in the stack were detected and isolated. Their three-dimensional position was determined using a centroid function. Finally, the minimum distance between two nuclei was calculated for the entire population of nuclei contained within 3D-CCMs.

### Cell Activity in 3D-CCM

#### Cell Viability

The viability was evaluated 28h after sample creation to determine the fate of cells embedded in the matrix. To extract cells from 3D-CCMs, samples were washed with 1X PBS and disaggregated in 1 mg/ml collagenase purchased from Clostridium histolyticum (Sigma-Aldrich^®^). After 30 min at 37°C, the collagenase was inactivated with complete medium and ethylenediaminetetraacetic acid solution (EDTA, Sigma-Aldrich^®^). Cells were stained with trypan blue and counted with a Luna automatic cell counter (Logos Biosystems©) which provides total, live and dead cells numbers, and so the cell viability. Viability study was performed 28 h after the 3D-CCM creation.

#### Cell Plating Efficiency

Cell plating efficiency (PE) was determined for each type of culture. For the 3D culturing, this was performed after cells recovering according to the protocol described in paragraph “Cell Viability.” In both cases, cells were plated in 100 mm diameter Petri dishes (Thermo Fisher) to obtain a density of 100 surviving cells per dish. The PE determined were 37% ± 11% (*n* = 8) for the cells extracted from the 3D cell culture and 61% ± 3% (*n* = 2) for the cells from the monolayer culture.

#### Cell Proliferation Assay

The metabolic activity of the cells was examined using a MTT [3-(4,5-dimethylthiazol-2-yl)-2,5-diphenyltetrazolium bromide)] assay. In this goal, 3D-CCMs were prepared to obtain ∼50 000 cells/sample. 3D-CCMs were successively exposed in a 96-well plate to 125 μl of tetrazolium dye MTT 3-(4,5-dimethylthiazol-2-yl)-2,5-diphenyltetrazolium bromide and incubated at 37°C for 4 h. Then, 125 μl of lysis buffer were added to dissolve the formazan crystals. Cellular viability and proliferation were measured at three time points after the sample creation: 12 h (Day 0), 33 h (Day 1) and 57 h (Day 2). Absorbance, proportional to the number of living and metabolically active cells, was quantified using a Glomax^®^ Microplate reader (Promega©) (absorbance 560 nm) and compared to the absorbance of a negative control treated with 200 μl of a 500 μmol/L toxic solution of menadione for 4 h.

#### Oxygen Tunability in 3D-CCM

The oxygen concentration in 3D-CCMs was tuned using the hypoxia workstation Hypoxylab^TM^ (Oxford Optronix©) where the concentration in oxygen (pO_2_) and in carbon dioxide (pCO_2_), as well as the temperature and the humidity can be set. Before adding the cell models, each well of a 96-well plate was filled with 320 μl of supplemented medium and maintained in the hypoxic workstation overnight to reach the level of the chosen O_2_ pressure. 3D-CCMs were then transferred to the wells inside the hypoxic device. The pO_2_ in 3D-CCMs was measured using the *in situ* sensor Oxylite^TM^ (Oxford Optronic©), which provides real-time information. A minimum of ten samples was considered for each measurement. A comparison with 2D was performed by measuring the pO_2_ value inside 5 flasks of monolayer cultures with 3 measurements for each. For each value, the conversion from mmHg to %O_2_ was carried out according to the following formula:

$$\%O_{2}=\frac{\left[\mathrm{mmHg}O_{2}\right]}{\left[\mathrm{atmospheric}\mathrm{pressure}\mathrm{in}\mathrm{mmHg}\right]/100}$$

### Nanoagent Monitoring in 3D-CCM

3D-CCMs were used to study the migration, uptake and toxicity of nanoagents. Gadolinium-based NPs called AGuIX^®^, which are currently being tested in the clinic and were provided by NH TherAguix (Lyon, France) were considered here. AGuIX^®^, composed of a polysiloxane matrix and gadolinium chelates, have a hydrodynamic diameter of 5 nm and a negative surface charge (Lux et al., 2018). They can be tagged with a Cyanine 5.5 fluorescent marker (AGuIX^®^ -Cy5.5) for microscopy experiments (Louis et al., 2005; Bridot et al., 2007). In this study, all the concentrations of AGuIX^®^ are expressed in the concentration of Gd^3+^, i.e., 1 mmol/L of Gd, which corresponds to 0.1 mmol/L of NPs.

#### Internalization Monitoring

The localization of nanoagents in 3D-CCMs was monitored by confocal microscopy according to the methodology described in Section “Confocal Microscopy.” From this perspective, the samples were prepared according to the protocol described in Section “Confocal Microscopy.” Briefly, 240 μl of 1 mmol/L AGuIX^®^-Cy5.5 was used to expose ∼350,000 HeLa cells/sample to the agents over 4 h. NPs emission was detected on a 655–740 nm spectral range upon excitation at 633 nm.

#### Uptake Monitoring

The mass of gadolinium contained in the samples was determined at the Ultra Trace Analyses Aquitaine (UT2A) Technological Center, Pau, France, using an Agilent 7800 ICP-MS (Agilent Technologies^®^). The objective was to determine the relative amount of AGuIX^®^ that was internalized by the cells and the amount of AGuIX^®^ that was trapped in the collagen. In this goal, six samples containing cells and collagen (3D-CCMs) were prepared. All samples were incubated in 240 μl of 1 mmol/L AGuIX^®^ solution for 4 h. The composition of the collagen-based model allowed for sample disaggregation according to the protocol given in Section “Cell Viability” in order to extract the cells and make a dosage of the gadolinium contained inside. Details of the uptake calculation are given in Supplementary Material, section B.

### Cell Response to Radiation Treatment in 3D-CCM

3D-CCMs were used to perform a clonogenic assay to evaluate treatment-induced cell death. However, sample disaggregation methodology could also be used to predict the efficacy of chemotherapy (Alberts et al., 1980; Liu et al., 2020), drugs (Law et al., 2020) or photothermal therapy (Zhang et al., 2019). Specifically, we investigated in this work the effect of AGuIX^®^ on cellular damage induced by gamma irradiation.

**Sample preparation.** For each irradiation experiment, fourteen 3D samples were prepared 36 h before irradiation according to the protocol described in Section “3D-CCM Production.” AGuIX^®^ were added to 3D-CCMs 18 h before irradiation at a concentration of 0.5 mmol/L in gadolinium. This concentration is known to be non-toxic to the cells (Lux et al., 2011; Porcel et al., 2014).

**Irradiation.** Irradiation was performed under atmospheric conditions with a 662 keV Cesium (^137^Cs) gamma source located at Institut Curie, Orsay (GSR-D1, RadeXp).

**Clonogenic assay.** Samples were then disaggregated according to the protocol given in Section “Cell Viability.” For each irradiation dose, three Petri dishes were prepared. Cells were then incubated for 15 days before fixation and staining in a solution of 50% methanol/50% methylene blue. Colonies consisting of at least 50 cells were counted. The survival fractions (SFs) were determined as the number of colonies counted divided by the product of the plating efficiency (PE) with the cell seeding.

**Statistical analysis.** A statistical analysis of the colony formation assay results was performed with the software package CFAssay for R (R Core Team, 2017), R: A Language and Environment for Statistical Computing. The F-test was performed based on the maximum likelihood (ML) method, which was described in 2015 by Braselmann and colleagues (Braselmann et al., 2015). The complete statistical analysis is provided in the Supplementary Material, section C.

## Results and Discussion

### Structural Characterization

#### Sample Thickness

3D-CCMs produced in 96-well plates have uniform radial size of 6.9 mm corresponding to the well diameter. However, due to the vertical compression exerted by the absorbers during the production process (see Section “3D-CCM Production”), a variability in the z direction was expected.

**FIGURE 1:**
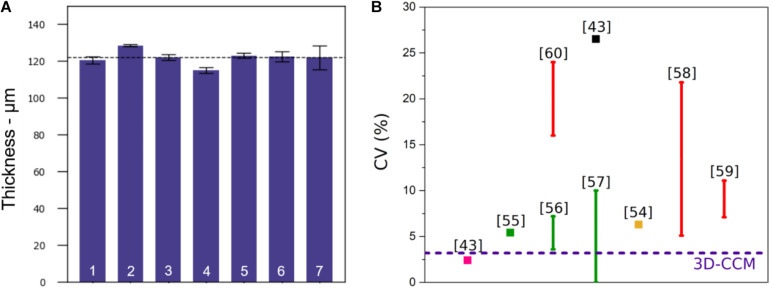
**(A)** Thickness of 3D-CCMs composed of HeLa cells (*n* = 7), **(B)** Comparison of the coefficient of variation (CV) obtained from the 3D-CCM thickness measurements (blue dashed line) with the CVs reported in the literature for spheroids of uniform sizes obtained by different methods: pellet culture (in pink), microwell arrays (in green), microfluidic devices (in red), hanging drop method (in black) or other (in yellow). The use of variation bars instead of square points illustrates the dependency of the CV to different parameters (cell seeding density, size of the well…).

The sample thickness was measured using the SHG collagen signal of each sample stack. The average thickness obtained on seven samples was 122 μm ± 4 μm, as shown in Figure 1A. The coefficient of variation (CV) (%), defined as the standard deviation divided by the mean height, was equal to 3.2%. This parameter was directly used to characterize the reproducibility of the sample preparation and compared with the values of the literature. A quantitative comparison of CV obtained with 3D-CCMs and the ones reported in different studies is illustrated in Figure 1B (Gong et al., 2015; Kang et al., 2015; Chen et al., 2016; Zanoni et al., 2016; Kwak et al., 2018; Sarkar et al., 2018; Shi et al., 2018; Thakuri et al., 2019).

Among the methods used to produce spheroids, three of them resulted in spheroid size uniformity: the pellet culture method, the hanging drop method and the spheroid preparation using microfluidic devices (Lin and Chang, 2008; Achilli et al., 2012; Benien and Swami, 2014; Cui et al., 2017). Zanoni et al. (2016) estimated the equivalent mean diameter of many of these spheroids and their associated standard deviations. The pellet culture method gives the best results in terms of size uniformity. With a spheroid diameter of 880 μm, the standard deviation of 21 μm leads to a CV of 2.4%. However, this approach is often neglected because its yield is low, the shear stress induces damage and the spheroids are difficult to manipulate (Lin and Chang, 2008; Achilli et al., 2012; Lazzari et al., 2017). With the hanging drop method, the variability is much higher than the one obtained for 3D-CCM: the standard deviation is 95 μm for a diameter of 359 μm (CV = 26.5%) (Zanoni et al., 2016). The use of patterned surfaces and microfluidics systems is an attractive strategy despite the tedious transfer of the created samples and the cost of the equipment (Lazzari et al., 2017). The samples generated in a controlled environment, present a uniform size which depends on the size of the wells (Kang et al., 2015; Lee et al., 2018) or on the initial cell seeding density (Gong et al., 2015; Chen et al., 2016; Kwak et al., 2018; Sarkar et al., 2018). The CV depends on these parameters as illustrated in Figure 1B.

For a concentration similar to ours (i.e., 1 × 10^6^ cells/ml), Kwak and coworkers obtained a CV of 22% (Kwak et al., 2018). So, the 3D-CCM CV is one of the lowest, which shows that the model is one of the most reproducible systems.

#### Cell Morphology and Distribution in 3D-CCM

**FIGURE 2:**
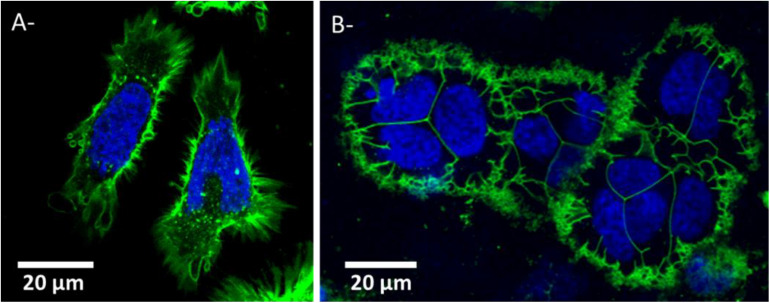
HeLa cells morphology in **(A)** 2D culture and **(B)** 3D cell collagen-based model. Nuclei and plasma membrane are stained in blue and green respectively. Panels **(A)** and **(B)** were obtained by confocal microscopy (Respective FOV of 82 μm × 84 μm and 123 μm × 83 μm and respective pixel size side of 0.186 μm or 0.312 μm).

The impact of cell morphology on the cell culture is shown in Figure 2 with HeLa cells cultivated in 2D (Figure 2A) and in 3D-CCM (Figure 2B). In the 3D model, the size of the cell was found smaller and its shape more spherical than in 2D cultures (Belli et al., 2017). The cell morphology in 3D-CCMs represents better the morphology of cells in tissues (Le et al., 2016). This effect of the culture conditions on the cell shape is explained by the presence of ECM, which exerts constraints on the cytoplasm, influences cell spreading and regulates tissue organization and cell fate (Muncie and Weaver, 2019). A cytoskeletal modification is induced in 2D cultures due to substrate adhesion. Note that a modification of morphology may induce a loss of polarity, which, in turn, may impact growth factor receptors and proliferation pathways (Yamada and Cukierman, 2007; Belli et al., 2017; Duval et al., 2017).

**FIGURE 3:**
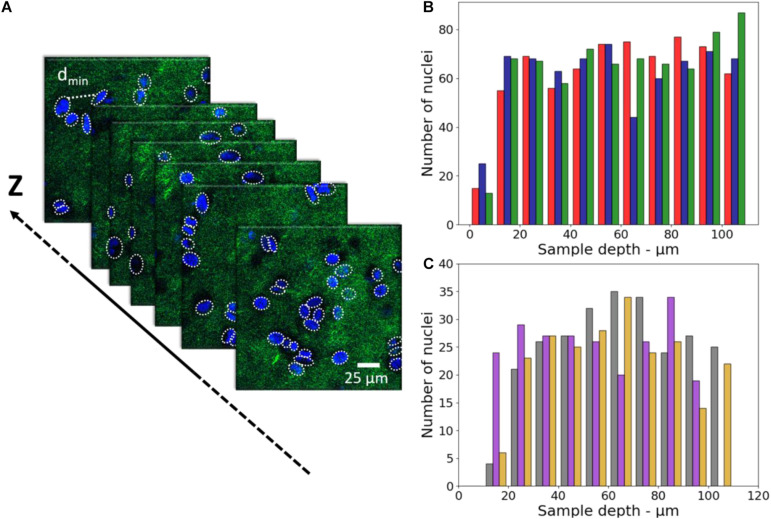
**(A)** 2PEF/SHG images of HeLa nuclei (in blue) embedded in the fibrillary collagen matrix (in green) (FOV of 350 μm × 350 μm, pixel size of 0.192 μm side), **(B)** Number of nuclei as a function of depth for HeLa in 3D-CCMs (*n* = 3, sample 1 in red, sample 2 in blue, sample 3 in green), **(C)** Number of nuclei as a function of depth for fibroblasts in 3D-CCMs (*n* = 3, sample 1 in gray, sample 2 in purple, sample 3 in yellow).

The characterization of the cell distribution on the sample is also a key issue to reproduce at best the spatial organization of cells in tissues. For HeLa cells, the concentration of 2.4–2.6 × 10^7^ cells/ml (see Section “3D-CCM Production”) was optimal to get a tight and homogeneous cell distribution. For comparison, we investigated the cell distribution with fibroblasts. In the latter case, a lower cell density was used to compensate the size difference between the two types of cells. As shown in the 2PEF/SHG images, Hela and fibroblasts cells presented the same homogeneous cell distribution in 3D-CCM. The images obtained with HeLa are shown only (Figure 3A). The number of nuclei obtained in each case, extracted from SHG/2PEF stack measurements, is plotted in Figures 3B,C.

These data show that the cells were evenly distributed along the *z*-axis and did not fall in the bottom of the sample despite gravity. As expected, more nuclei were found with Hela cells because of the higher density used in this experiment.

**FIGURE 4:**
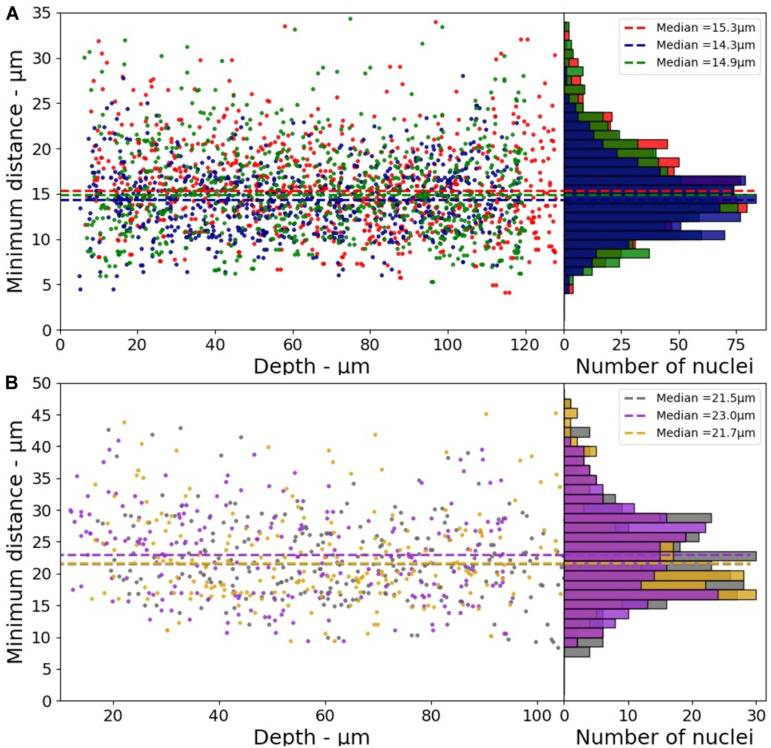
Minimum distance between two nuclei as a function of depth for 3D-CCMs composed of **(A)** HeLa cells **(B)** Fibroblasts (*n* = 3).

The minimum distance between two nuclei was calculated to characterize the cell distribution in the 3D model (x-y-z directions). The median values of the minimum distances obtained from three different samples are reported in the Figure 4. These values vary from 14.3 to 15.3 μm with a standard deviation of 0.5 μm (∼3.3%) for HeLa, and from 21.5 to 23.0 μm with a standard deviation of 0.8 μm (∼3.7%) for the fibroblasts.

These values are consistent with that reported in the literature. Internuclear distances of 4–30 μm are reported for the tibialis anterior muscle in mice (Stroud et al., 2017). Another study based on hematoxylin and eosin *(H&E)-*stained images of mouse brain showed that the internuclear distance is lower than 25 μm (Li et al., 2018). Yi et al. (2017) reported a method of automatic extraction of cell nuclei from *H&E-*stained images of human lung tumors in which the order of magnitude of the nucleus-nucleus distance is approximately 10 μm. This distance distribution between nuclei shows that cells are homogeneously distributed in 3D-CCMs, thus faithfully mimicking the distribution of cells in tissues.

### Cell Activity

**FIGURE 5:**
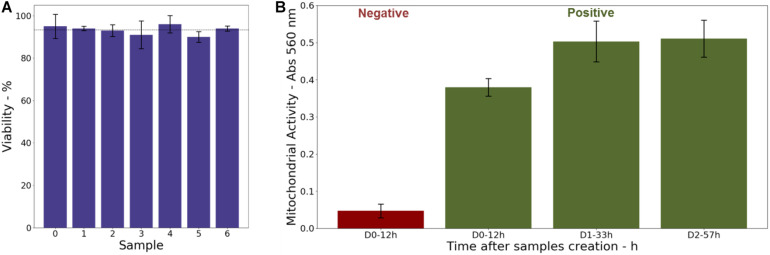
**(A)** Cell viability in 3D-CCMs measured 28 h after model creation. **(B)** Evolution of the mitochondrial activity in 3D-CCMs (in green) quantified by MTT assay as function of the time in the sample. The negative control is shown (in red).

As shown in Figure 5A, a viability higher than 90%, with an average value close to 93%, was observed for all the samples. It indicates that cells stayed alive in 3D cultures for several days. This is confirmed, for instance, by the cell division process observed in the confocal image (Figure 2B) acquired 48 h after the sample preparation.

In parallel, a MTT assay was performed to assess the mitochondrial activity of the cells embedded in the collagen matrix as function of the time after the 3D-CCMs creation. As illustrated in Figure 5B, the results (in green) were compared to a negative control (in red) composed of dead cells. A strong mitochondrial activity was observed. It proves that the cell metabolism is not impacted even after 57 h in 3D-CCM. The activity increase attests of the ability of the cells to proliferate. Between the first and the second day (i.e., 11 h), the cellular metabolic activity increased by more than 30%. Between the 2nd and the 3rd day, it remained stable, which reflects a slow-down of the cell proliferation. This finding agrees with the literature where a reduced proliferation is observed for a variety of cell lines in 3D cultures (Maria et al., 2011; Adcock, 2015).

### Oxygen Control

The oxygen concentration in 3D-CCMs was tuned so as to mimic various tissue environments. In 2D samples, the cells are typically maintained in incubators to reproduce normoxic conditions in a controlled atmosphere of 37°C, 5% CO_2_, and 21%-160 mmHg O_2_. In this condition, pO_2_ is close to 143 mmHg ± 2 mmHg (19.4% ± 0.2%). In healthy tissues, pO_2_ is of the order of 50 mmHg (Wion et al., 2008). In 3D-CCMs, pO_2_ measured after one night in the incubator (37°C, 5% CO_2_, 21% O_2_) is 112 mmHg ± 12 mmHg (14.7% ± 1.5%). This value is close to pO_2_ observed in physiological conditions (i.e., 100 mmHg (13.5%) in body lung alveoli (McKeown, 2014)). The pO_2_ depletion observed in 3D cultures is attributed to the O_2_ consumption by the cells and not to reduced oxygen diffusion in the matrix (Cheema et al., 2012; Ehsan and George, 2013; Sarem et al., 2019). Moreover, pO_2_ was determined using T-25 flasks for 2D samples and 96 wells plates for 3D-CCMs. Differences of depth from the medium surface, medium volume and cell density are expected to affect pO_2_ (Oze et al., 2012; Zhang et al., 2016; Place et al., 2017).

**FIGURE 6:**
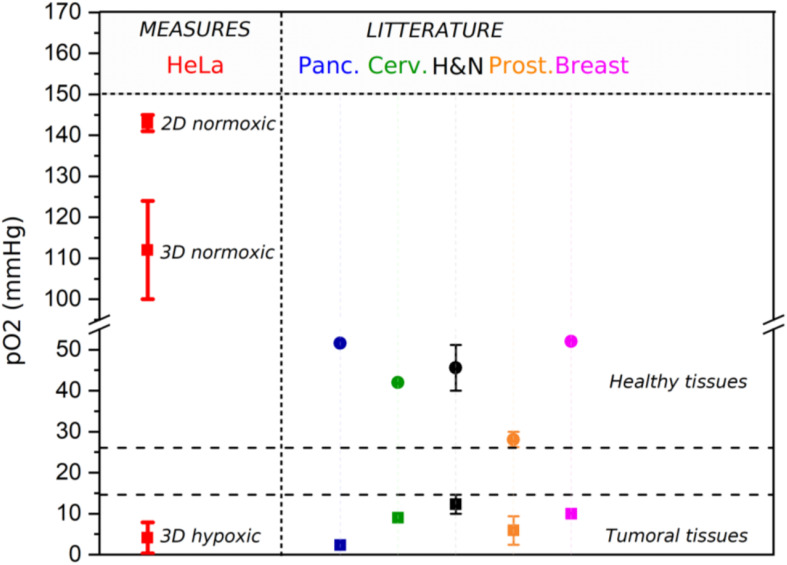
pO_2_ measured in 3D and 2D samples (red bold bar) (*n* = 10). It is compared to pO_2_ reported in the literature (McKeown, 2014) for tumoral (square) and healthy tissues (circle), such as pancreas (blue), cervix (green), head & neck (black), prostate (orange) and breast (pink). For the values reported from literature, an error bar was represented when several studies were considered.

We investigated the oxygen control in 3D-CCMs incubated in different atmospheres. Exposure of 3D samples to a controlled atmosphere containing 18 mmHg of oxygen (2.3%) allowed to artificially reproduce the oxygenation conditions in tumors. We also found that pO_2_ increased with the time of exposure of 3D-CCM to hypoxic conditions. pO_2_ varied from 0.6 ± 0.3 mmHg after 1 h exposure, to 1.3 ± 1.4 mmHg after 3 h and to 5.6 ± 2.3 mmHg after 5 h. Values below 8 mmHg (1%) are associated to a “pathological hypoxia” (McKeown, 2014). Figure 6 summarizes the pO_2_ values measured in 2D and 3D cultures in Hela cells exposed to different oxygen conditions (normoxic and hypoxic). They are compared to values reported in the literature for various tissues.

This experiment demonstrated that 3D-CCM is fully adapted to study the impact of treatments on cells growing in hypoxic tissues such as tumors.

### Evaluation of Drugs/Nanoagents Uptake in Living Cells

**FIGURE 7:**
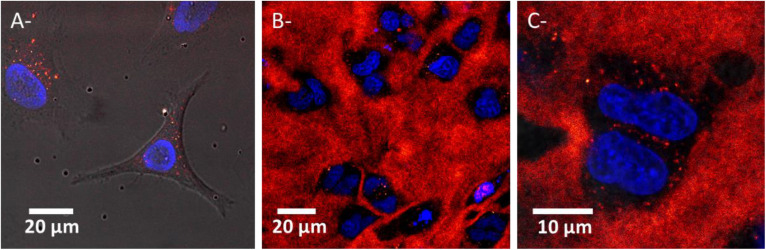
Confocal microscopy images of HeLa cells exposed to AGuIX^®^ in **(A)** a 2D culture (FOV of 110 μm × 101 μm, pixel size of 0.229 μm side) and **(B,C)** 3D-CCMs (FOV of 140 μm × 140 μm and 41 μm × 41μm, pixel size of 0.273 and 0.080 μm side). Each image results from superimposition of the transmission and fluorescence images. Nuclei and NPs are labeled in blue and red respectively. The black holes correspond to cells (absence of collagen).

The cellular uptake of AGuIX^®^ in Hela cells embedded in 3D-CCMs was followed using confocal microscopy (Figure 7). The images show the NPs infiltration in the collagen matrix (Figure 7B) and cells (Figure 7C) after incubation with 0.5 mmol/L AGuIX^®^ for 18 h. We observe that the NPs are homogenously distributed in the collagen matrix. Although the collagen is a natural barrier that hampers the transport of nanoagents (Belli et al., 2017) and limits the internalization of nano-objects (Fallica et al., 2012; Costa et al., 2016), we found AGuIX^®^ aggregates in cells, in the cytoplasm exclusively. No nanoparticles were observed in the nucleus, as already reported in 2D cultures (Figure 7A) (Porcel et al., 2014; Štefančíková et al., 2014).

The quantification of nanoagents internalized in cells and trapped in the collagen was performed by ICP-MS. In this perspective, 3D-CCM were disaggregated (see Section “Cell Viability”). The results are presented in Table 1. This measurement indicates that, in these conditions of incubation (see Section “Uptake Monitoring”), 1.2 × 10^13^ NPs penetrated in 3D-CCMs, from which 5.9 × 10^11^ were internalized in the cells (20 times less). It corresponds to internalizations close to 0.1% of NPs in 3D-CCMs and 0.004% in the cells. This result is in agreement with the microscopy observations presented above.

**TABLE 1:** Quantification of AGuIX^®^ determined by ICP-MS in (A) collagen + cells (3D-CCMs) and (B) cells extracted from 3D-CCM.

	COLLAGEN + CELLS 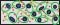	CELLS 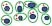
Mass of Gd (μg)/sample	0.031 ± 0.005	0.002 ± 0.001
NPs (#)/sample	1.2 E + 13	5.9 E + 11
Concentration of Gd per cell (pmol/L per cell)	−	0.056
Uptake/sample (%)	0.1	0.004

This experiment confirms that 3D-CCM is suitable for investigating nanoagents uptake in tissue-mimicking samples. In particular, it demonstrates that, at even low amounts, AGuIX^®^ diffuses through the collagen and is partially engulfed in cells embedded in the matrix.

### Quantification of Radiation Effect on Cell Survival

**FIGURE 8:**
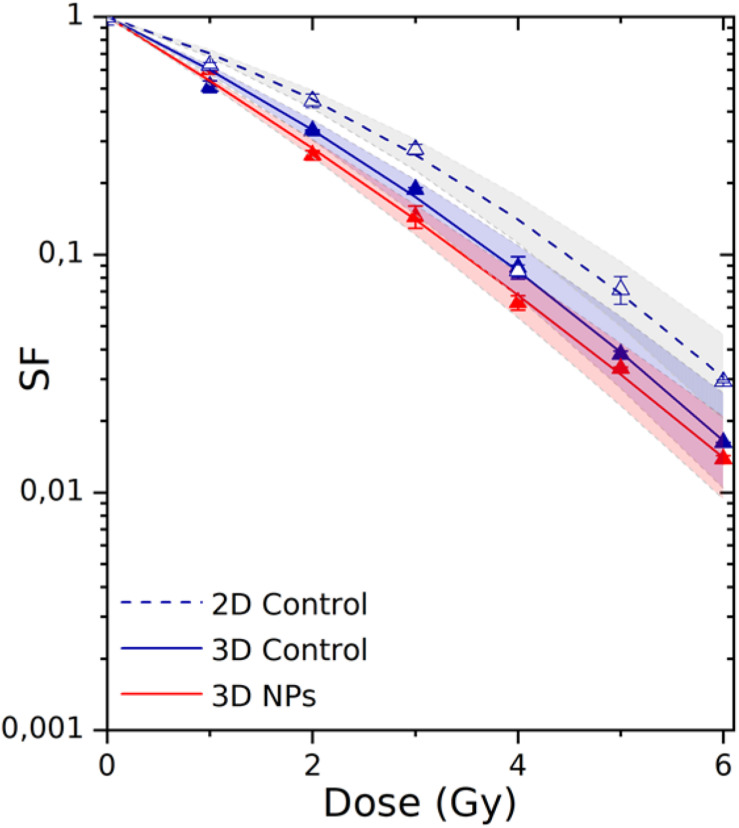
Survival fractions of HeLa cells irradiated in 2D culture (dashed curve/empty triangles) and in 3D-CCMs (solid curve/solid triangles). The influence of AGuIX^®^ on radiotoxicity was investigated using 3D-CCMs (red curve). The theoretical uncertainties (area) were determined according to the standard deviations of the α and β parameters. F-statistical tests, based on the maximum likelihood method (see details in Supplementary Material, section C), were performed to compare 2D and 3D models, and, models with or without AGuIX^®^. The differences were significant with a *p* value of 1.099e-07 (*p* < 0.05) and 0.03 (*p* < 0.05).

The robustness of 3D-CCMs to study external treatments effects on cells was evaluated by investigating the effects of gamma rays on cell survival and, also, the influence of nanoagents on the radiotoxicity. In this perspective, the samples were disaggregated (see Section “Cell Viability”) after the irradiation to proceed with clonogenic assay analysis. This method is the gold standard of radiobiology to quantify the effect of radiation on cell death and proliferative loss. The survival curves are presented in Figure 8.

The cell response function was simulated using a linear quadratic law, where α is associated with the contribution of the directly lethal lesions induced in the cell and β with the accumulation of additive sublethal lesions (Tubiana et al., 1986). The parameters of the theoretical fits are given in Table 2.

**TABLE 2:** Calculated radiobiological coefficients.

*SF*(D) = *e*^−(α*D* + β*D*^2^)^	α (Gy^–1^)	β (Gy^–2^)	α /β (Gy)	*R*^2^	SER_2Gy_ (%)	DEF_10%_
Control 2D	0.31 ± 0.03	0.05 ± 0.01	7	0.99	N.A	N.A
Control 3D	0.48 ± 0.03	0.03 ± 0.01	16	>0.99	N.A	N.A
AGuIX^®^ 3D	0.60 ± 0.03	0.02 ± 0.01	30	>0.99	16	1.09

*Dependency R^2^ values are reported in the table.*

#### Comparison of the Results Obtained in 2D and 3D Models

The 2D culture is the primary reference model used in radiobiology. Thus, we first compared the results obtained in 2D culture and 3D-CCM. The survival fraction (SF) of the cells irradiated in 2D cultures decreased exponentially with increasing irradiation doses, which corresponds to previous studies (Porcel et al., 2014; Štefančíková et al., 2014). We show here that a similar trend is observed for cells irradiated in 3D-CCMs. α is close to 0.48 Gy^–1^ for the 3D culture and 0.31 Gy^–1^ for the 2D culture. It demonstrates that the directly lethal damage induced by radiation are more important for cells embedded in 3D-CCM. On the contrary, the α value is constant (0.05 Gy^–2^) in the two cases. In total, the α/β ratio, a parameter that is representative of the cell radiosensitivity, is about two times higher for cells in collagen.

This result is different from other studies where cells in 3D models (spheroids for instance) generally present a higher resistance to radiations (Doctor et al., 2020). Contrary, it is consistent with results published on the irradiation of cervical carcinoma cells incorporated in hydrogels based on collagen I, where the effect of radiation was found higher in 3D than in 2D models (Topsch et al., 2007; Walenta and Mueller-Klieser, 2016). This observation can be attributed to difference in cell cycle between 2D and 3D cultures. In 3D cultures in particular, the radiosensitivity of cells is higher because the cells stay in a prolonged arrest in the G2/M-phase, the most sensitive to radiations. In addition, the doubling time of cells in 3D samples (61.2 h) is three times higher than the doubling time cells in 2D monolayers (17.3 h). Thus, the number of colonies counted in the clonogenic assay (min 50 cells) may be underestimated for the 3D cultures.

#### Application of 3D-CCM to the Evaluation of NPs Induced Amplification of Radiation Effects

3D-CCM was used to evaluate the influence of nanoagents on radiation effects. We investigated the response of the HeLa cells treated with AGuIX^®^ and by radiation. As shown in Figure 8, the decrease in cell survival was stronger in the presence of AGuIX^®^. It demonstrates that the exposure to NPs of cells embedded in 3D-CCMs amplifies efficiently radiation effects.

The amplification efficiency of NPs is commonly quantified using two parameters, namely the radiation Sensitizer Enhancement Ratio (SER) and the Dose Enhancement Factor (DEF). Their calculation is detailed in the Supplementary Material, section C. As reported in Table 2, the survival fraction at 2 Gy was reduced by 16% for cells incubated with AGuIX^®^ in 3D-CCMs. Interestingly, the induction of directly lethal damage doubled in the presence of NPs.

These results demonstrate for the first time in a tissue-like model, that AGuIX^®^ improve the quality of radiotherapy treatments.

### Conclusion

This work demonstrates the advantage of the 3D cytoarchitecture and collagen-based cell model to investigate the impact of various cell treatments. The production of 3D-CCMs, mimicking the microenvironment of cells in tissues, requires minimal material. It is a rapid and robust method which is adaptable to several cell lines.

We found that the preparation of the samples is highly reproducible. The size of the models is constant and the distribution of cells is homogeneous. Thanks to the optical properties of the collagen matrix, label free multiphotonic microscopy can be used to characterize the samples and monitor the internalization of agents (such as gadolinium NPs) in living cells. Another major advantage of the model is that the oxygen concentration may be tuned so as to reflect various architectures and physiologies of tissues.

Many tests, frequently used with monolayer cultures, remain directly applicable to the 3D cultures such as metabolic activity assays (MTT). Furthermore, the cells are easily and rapidly recovered by disaggregation of 3D-CCMs. Thus, the impact of various treatments on cells may be evaluated using single cell experiments. As an example, we successfully addressed the toxicity of external agents (gadolinium NPs) and the effect of radiation treatment on cancer cells in this tissue-like sample.

In summary, 3D-CCM is an advantageous *in vitro* model that may be applied to rapidly assess the effect of novel therapies in conditions more realistic than 2D cell cultures. This promising model brings *in vitro* experiments one stage closer to the *in vivo* application, without ethical and financial constraints of animal experiments.

## Data Availability Statement

The original contributions presented in the study are included in the article/Supplementary Material, further inquiries can be directed to the corresponding author/s.

## Author Contributions

PMau, EP, and SL conceived the project. PMau and EP performed irradiation experiments and proposed experimental data interpretations. PMah, M-CS-K, PMau, and EP performed the microscopy experiments. PMau and AM did the image analysis and Python program development. FL and OT designed and prepared AGuIX© nanoparticles. EP and SL obtained the findings. SL coordinated the project. All authors contributed to writing the manuscript and approved the final version.

## Conflict of Interest

FL and OT must disclose the patent WO2011/135101. This patent protects the AGuIX^®^ nanoparticles described in this publication and their administration via the airways. FL and OT are employees of NH TherAGuIX^®^ which is developing the AGuIX^®^ nanoparticles. FL and OT possess shares of this company. The remaining authors declare that the research was conducted in the absence of any commercial or financial relationships that could be construed as a potential conflict of interest.
