# Supplementary material for: Rapid Evaluation of Novel Therapeutic Strategies Using a 3D Collagen-Based Tissue-Like Model
Source: Front Bioeng Biotechnol. 2021 Feb 16;9:574035. doi: 10.3389/fbioe.2021.574035 (PMC7929985; doi:10.3389/fbioe.2021.574035)
Supplement: Supplementary file 1 [file Presentation_1.pdf]

1. Supplementary Materials

A- Segmentation and analysis of nuclei

The analysis was performed using Python 3.7, whose code and data are available online on Zenodo (DOI: 10.5281/zenodo.3814365). Cell nuclei were distinguished using a threshold method. Due to absorption and scattering, the excitation intensity decreases with depth. Thus, stack images present different intensity statistics. This variation was considered with an adaptive threshold **t**, which linearly varies with the **σ** standard deviation of individual image stacks (t = a***σ** + b). The slope of this linear threshold was approximately 1 and its intercept was taken around the median value of the global image.

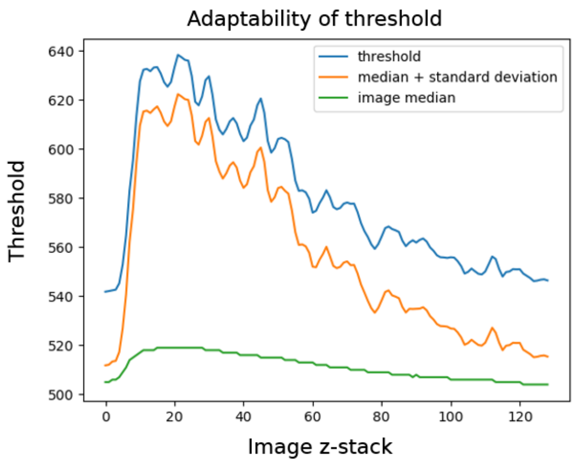

Figure 1. Varying the threshold with imaging depth. Green, orange and blue curves respectively indicate the median of the image at each stack, the sum of the median and the standard deviation, and the adaptive threshold. In this example, the threshold was 1.0*std_dev + 535.

Before application of the threshold, each stack was smoothed by a local median filter. Once the 3D binary image was generated with this adaptive threshold, a binary opening operation was performed to erase small erroneous spots and to maintain wide cell-related parts. Then, the

measure.label function from skimage distinguished individual nuclei in three dimensions, based on the binary image.

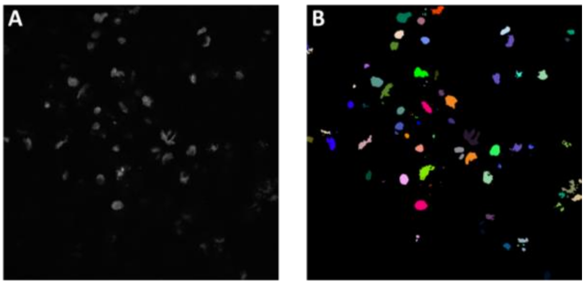

Figure 2. Example of the clustering of individual cells in 2D: A- Cell image at a fixed stack, B- Resulting clustering image where each color indicates a different cell.

For each nuclei cluster, we then extracted the size, centroid and global intensity parameters. The distance between each nucleus centroid was calculated to extract the nucleus-nucleus minimum distance using the nearest neighbor python algorithm. The global workflow is described in **Figure 3.**

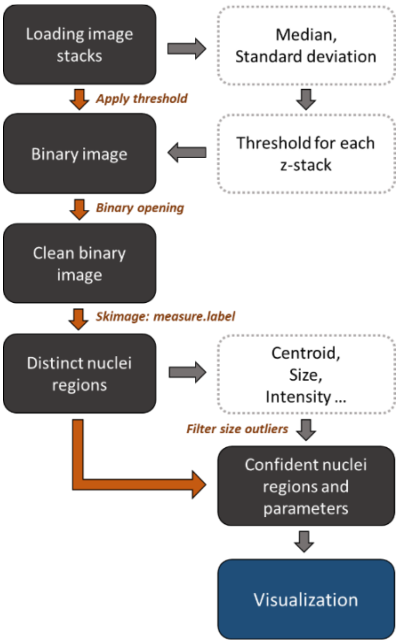

Figure 3: Global Python code workflow. The adaptive threshold is applied to generate a 3D binary image. This image is cleaned via an opening, and parameters are extracted for individual regions. After a final filtering of outliers, plots and histograms are generated.

B- Uptake calculation

The number of NPs per sample was calculated from the mass value using the equation (2):

$$\frac{NPs}{sample} = \frac{m_{Gd}.N_A}{n.M_{Gd}.(atoms_{Gd}/NP)} \qquad (2)$$

where m_{Gd} is the mass of Gd in g.mol^{-1} determined by ICP-MS,

n is the number of samples, and here, n=3 because we prepared 3 samples per condition (see section **Erreur ! Source du renvoi introuvable.**)

N_A is Avogadro's number equal to 6.02.10^{23} mol^{-1},

M_{Gd} is the Gd molar mass equal to 157.25 g.mol^{-1}, and

$(atoms_{Gd}/NP)$ represents the number of Gd atoms per NP, *i.e.,* 10 for the AGuIX®.

To calculate the uptake, we determined N_{NPs}, the quantity of NPs available during the 4 h of incubation given by the equation (3):

$$N_{NPs} = \frac{C_{incub}.V_{incub}.N_A}{(atoms_{Gd}/NP)} \qquad (3)$$

where C_{incub} is the incubation concentration of NPs equal to 1.10^{-3} mol.L^{-1} (see section **Erreur ! Source du renvoi introuvable.**.),

V_{incub} is the incubation volume, *i.e.,* 240 µl according to the protocol given in section **Erreur ! Source du renvoi introuvable.**, and uptake is determined by the ratio of (2) to (3).

C- Clonogenic assay

Statistical test. A test for the comparison of cell survival curves was performed using the package CFAssay implemented in R software. Colony numbers are discrete values following the Poisson distribution. The maximum-likelihood (ML) method was chosen and the R-function glm (generalized linear model) was used. The workflow of this open access package is divided

into three steps: (1) data input and double-check, (2) separate calculation of cell survival curves for each of the two conditions, (3) comparison test of the curves for the two experiments. [53,84]

First, the survival curves of the two conditions (with and without NPs) were independently plotted using the *cellsurvLQfit* function, and plating efficiencies were fitted as intercepts. The ML method gives the α and β values (that we compared with those given by Origin) and the dispersion parameter (d.p).

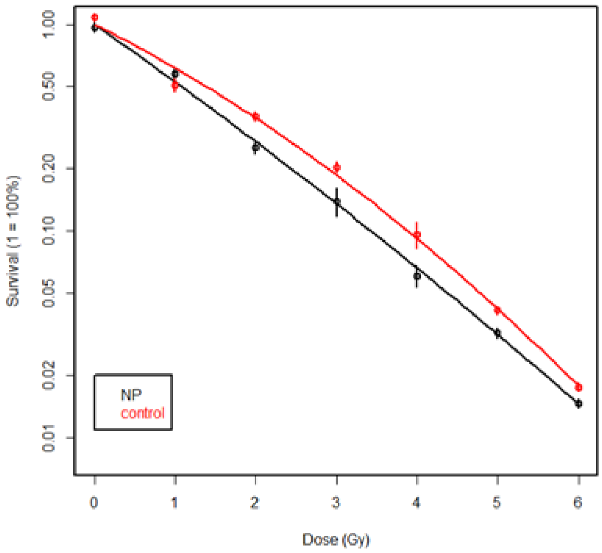

	α (Gy^{-1})	β (Gy^{-2})	d.p
control	0.45±0.05	0.04 ± 0.01	1.97
3D	0.63 ± 0.05	0.01 ± 0.01	1.5

The dispersion parameter was compared with the value of 9.00, which corresponds to 3 Poisson standard deviations, and shows low variability in the data. The function *cellsurvLQdiff* was used to compare the control and the AGuIX® fits using the ANOVA F-test. This statistical test is composed of two models. Model 1 corresponds to the null hypothesis, which is the hypothesis to reject (*i.e.*, “There is no difference between the two fits”). The curve is fitted to the total cell survival. Parameters α and β are independent of the two curves for 2D and 3D, while for model 2, the curves are fitted separately. The F value obtained after the F-test was 3.93 with a p value of 0.030 ($p>0.05$). This p-value, which is the probability that the difference between the residual data scatter of model 1 compared with that of model 2 occurs by chance, indicates a significant difference.

Efficiency. The efficiency of AGuiX® to amplify gamma radiation was calculated using two indicators obtained from the survival curves. The radiation sensitizer enhancement ratio (SER) is indicative of the radiation effect induced by the presence of AGuIX®. This parameter is defined at a dose point, specifically 2 Gy, which is the reference dose/fraction in conventional radiotherapy treatments.

$$SER_D(\%) = \frac{SF_D^c - SF_D^N}{SF_D^c} \qquad (4)$$

where SF_D^c and SF_D^N are the survival fractions at the D dose for the control sample and the sample incubated with AGuIX®.

The dose enhancement factor (DEF) is the ratio between the radiation dose needed to achieve a certain survival fraction in the control, D_{SF}^c, and the radiation dose needed to obtain the same biological effect in the presence of AGuIX®, D_{SF}^{NP}. This indicator is commonly calculated for 10% of SF.

$$DEF_{SF} = \frac{D_{SF}^c}{D_{SF}^{NP}} \qquad (5)$$
